# Gastroprotective and Antioxidative Effects of the Traditional Thai Polyherbal Formula Phy-Blica-D against Ethanol-Induced Gastric Ulcers in Rats

**DOI:** 10.3390/nu14010172

**Published:** 2021-12-30

**Authors:** Sineenart Sanpinit, Piriya Chonsut, Chuchard Punsawad, Palika Wetchakul

**Affiliations:** 1 Department of Applied Thai Traditional Medicine, School of Medicine, Walailak University, Thasala, Nakhon Si Thammarat 80160, Thailand; sineenart.sn@wu.ac.th (S.S.); piriya.ch@wu.ac.th (P.C.); 2 Research Center in Tropical Pathobiology, Walailak University, Nakhon Si Thammarat 80160, Thailand; chuchard.pu@wu.ac.th; 3 Department of Medical Science, School of Medicine, Walailak University, Thasala, Nakhon Si Thammarat 80160, Thailand

**Keywords:** herbal formula, gastroprotective effect, antioxidant activity, oxidative stress, ethanol-induced gastric ulcer, Phy-Blica-D

## Abstract

Phy-Blica-D is a traditional Thai polyherbal formula that has reduced oxidative stress in non-communicable diseases. However, evidence supporting the gastroprotective effects of Phy-Blica-D has not been previously reported. Therefore, this study aimed to evaluate the gastroprotective effects of Phy-Blica-D against gastric ulcers in rats and investigate the potential underlying mechanism. To estimate the possible mechanisms of action, we examined the levels of oxidative stress markers, such as reactive oxygen species (ROS) and malondialdehyde (MDA), as well as antioxidant enzymes, including catalase (CAT), superoxide dismutase (SOD), and glutathione (GSH). According to our results, rats treated with only 80% ethanol (vehicle group) exhibited significant increases in their ulcer area and ulcer index (UI). Moreover, the levels of ROS and MDA markedly increased in the vehicle group compared with the normal control group. Daily oral administration of Phy-Blica-D (500 and 1000 mg/kg) for 7 days not only significantly decreased the ulcer area and UI, but also remarkably decreased the ROS and MDA levels in gastric tissue. Gastric ulcers induced by ethanol had significantly decreased antioxidant enzyme activities (CAT and SOD) and non-enzymatic antioxidant (GSH), whereas pretreatment with Phy-Blica-D significantly improved the activities of CAT, SOD, and GSH. Moreover, after exposure to ethanol, the rats exhibited a significantly increased level of inducible nitric oxide synthase (iNOS), which was reduced after treatment with Phy-Blica-D. These findings suggest that Phy-Blica-D potentially exerts its gastroprotective effects by suppressing oxidative stress and stimulating antioxidant enzymes, which is one of the causes of destruction of cell membranes, and it is involved in the pathogenesis of acute gastric ulcers induced by ethanol.

## 1. Introduction

Gastric ulcers are one of the most common diseases of the digestive system. The pathophysiology of this disease is a multifactorial process that is caused by an imbalance of gastric mucosa-protecting (pepsin) and gastric mucosa-destroying factors (acid) and is induced by infection, smoking, stress, the prolonged use of nonsteroidal anti-inflammatory drugs (NSAIDs), and excessive alcohol ingestion [1]. Ethanol is a harmful agent associated with multiple pathologies and can be orally applied to experimental animals to create acute gastric lesions [2]. Ethanol-induced gastric ulcers result from disruption of the gastric mucosa, which increases mucosal permeability and bleeding. White blood cells, such as neutrophils, infiltrate the site of gastric injury and cause the overproduction of reactive oxygen species (ROS) and other mediators of inflammation, resulting in oxidative damage and damage to cells [1,2]. ROS are the main mediators of oxidative stress and decrease the activity of antioxidant enzymes (superoxide dismutase—SOD and catalase—CAT) and non-enzymatic antioxidant (glutathione; GSH) [3]. These are some of the causes of destruction of cell membranes and are involved in the pathogenesis of acute gastric ulcers induced by ethanol.

In addition, a previous study reported that oxidative stress during gastric inflammation induced by ethanol stimulates lipid peroxidation, as indicated by elevated malondialdehyde (MDA) levels in gastric tissues [4]. Nuclear factor kappa B (NF-κB) activation occurs during mucosal inflammation induced by gastric ulcer disease. It has been reported that NF-κB controls the generation of proinflammatory cytokines, such as interleukin 1beta (IL-1beta), interleukin-6 (IL-6), tumor necrosis factor-alpha (TNF-alpha), and inducible nitric oxide synthase (iNOS), which are involved in inflammatory reactions [5,6]. Several modern drugs, such as antacids, antibiotics, H2 receptor antagonists, and proton-pump inhibitors (PPIs), are widely used for the treatment of gastritis [7]. Omeprazole (OMZ), a PPI, has been reported to exert a gastroprotective effect in numerous published studies [7,8,9,10,11]. However, long-term treatment with modern drugs can cause adverse effects [12]. The adverse effects of PPIs can be divided into two types: those unrelated to acid inhibition and those related to acid inhibition. The adverse events unrelated to acid inhibition include acute interstitial nephritis, chronic kidney disease, and collagenous colitis. The adverse events related to acid inhibition are gastric carcinoid tumor, gastric fundic mucosal hypertrophy, changes in the gut microbiome, small intestinal bacterial overgrowth, gastric fundic gland polyps, and gastric cancer [13]. Due to the adverse effects of modern drugs used for the treatment of gastric ulcers, alternative treatments are needed.

Herbal medicines are alternative medicinal treatments that have different gastroprotective mechanisms, including stimulation of mucosal proliferation, inhibition of acid production, and antioxidant properties [14,15]. Traditional Thai herbs and their ingredients have been used for medicinal purposes since ancient times. Some traditional Thai herbs are used for their antioxidant, anti-gastric-ulcer, and anti-inflammatory properties [16].

Phy-Blica-D is a traditional Thai polyherbal infusion used as a rejuvenating formula. It was developed from the original formula, Phy-Blica-O or THP-R016, which has a high content of total phenolics and flavonoids and exhibits strong antioxidant activity. However, Phy-Blica-O has a strong bitter taste and unpleasant odor. According to a recent study, modification of Phy-Blica-O yielded high sensory acceptability scores. Phy-Blica-D, one of the modified formulas, scored the highest in taste and overall acceptability. Moreover, it has excellent antioxidant activity, reduces oxidative stress in vitro, and is nontoxic at a dose of >300 mg/kg body weight (BW)/day [17]. Phy-Blica-D has been reported to contain phytochemical compounds, including 6-galloylglucose, 1-O-galloylglycerol, fertaric acid, vanilpyruvic acid, (2S)-5,7,3′,4′-tetrahydroxyflavanone 6-C-glucoside, naringerin, agecorynin B, castavinol, chalconaringenin 2′-rhamnosyl-(1→4)-xyloside, beta-rhodomycin, sericoside, alliosterol 1-(4″-galactosylrhamnoside) 16-galactoside, licorice saponin A3, asparasaponin II, licorice saponin G2, betavulgaroside II, glycyrrhizic acid, and 6-gingerol [17]. Moreover, a literature review demonstrated that the phytochemical compounds of Phy-Blica-D herbal component are various, but the main chemicals are phenolic, flavonoid, alkaloids, glycosides, tannin, and triterpenoid, as shown in Table 1 [18,19,20,21,22,23,24,25,26,27,28,29,30,31,32,33,34,35].

A previous study demonstrated that Phy-Blica-D is safe and reduces oxidative stress in the context of noncommunicable diseases. Gastric ulcers are caused by oxidative stress and are related to dysfunction of antioxidant enzymes [36]. Therefore, the anti-gastric-ulcer activity of Phy-Blica-D has attracted considerable interest. The aim of this study was to evaluate the gastroprotective effects of Phy-Blica-D against ethanol-induced gastric ulcers in rats and the potential underlying mechanism.

## 2. Materials and Methods

### 2.1. Chemicals and Reagents

All materials and reagents were obtained from Sigma (Sigma-Aldrich Pty Ltd., Darmstadt, Germany), and the CAT, SOD, GSH, ROS, MDA, and iNOS activity measurement kits were purchased from Sunlong Biotech Co., Ltd. (Sunlong Biotech Co., Ltd., Hangzhou, China). OMZ (Berlin Pharmaceutical Industry, Bangkok, Thailand) was used as a reference antiulcer drug.

### 2.2. Plant Collection and Extraction

The medicinal components of Phy-Blica-D, which are shown in Table 1, were purchased from a local licensed medicinal plant store, Triburi Orsot (Triburi Orsot, Songkla, Thailand). A total of 17 Phy-Blica-D infusions were prepared by adding 1 tea bag to 120 mL of freshly boiled water at 98 ± 2 °C and brewing for 3 min without stirring. The Phy-Blica-D sample was filtered using filter paper (Whatman No. 1). The extracts were completely dried in a lyophilizer and stored at −20 °C until use. The percent yield of raw materials was 14.16%.

### 2.3. Animals

Adult male Wistar rats weighing 180–200 g were purchased from Nomura Siam International Co., Ltd., Bangkok, Thailand. They were maintained under standard environmental conditions: light–dark cycle, 12–12 h; temperature, 22 ± 2 °C; humidity, 40–45%. The rats were allowed ad libitum access to food and water during the experimental period.

### 2.4. Ethical Approval Statement

The experimental protocols performed in this study were designed in accordance with Good Laboratory Practice and approved by the Animal Ethical Committee of Walailak University, Thailand (WU-AICUC-64-003).

### 2.5. Experimental Procedure

#### 2.5.1. Determination of In Vitro Antioxidant Activity

The oxygen radical antioxidant capacity (ORAC) assay was carried out to determine the antioxidant activity of Phy-Blica-D extract against peroxyl radicals generated by thermal homolysis of 2,2′-azobis (2-amidinopropane) dihydrochloride (AAPH) according to the method of Wetchakul et al. [37]. In addition, a superoxide radical (•O^2^−) scavenging assay was performed to evaluate the reduction in nitroblue tetrazolium (NBT) according to the method described by Chanthasri et al. [29]. Catechin was used as the positive control of the NBT assay.

#### 2.5.2. Ethanol-Induced Gastric Ulcers in Rats

The rats were randomly assigned to one of five groups (*n* = 6), as shown in Table 2. All rats were orally administered the appropriate treatment once daily for a period of 7 days. One hour after the final administration, all rats except those in the normal control group were given 80% ethanol orally (1 mL/kg BW) to establish an acute gastric damage model according to a previously reported method [38] with some modifications. Four hours after ethanol administration, the rat stomachs were removed.

#### 2.5.3. Gastric Juice Acidity Measurements

The acidity of the gastric juices of each rat was evaluated individually by immersing a pH indicator strip in the gastric juices immediately after opening of the stomach. The results are expressed as a pH value.

#### 2.5.4. Macroscopic Gastric Lesion Evaluation

The rat stomachs were opened along the greater curvature, rinsed with normal saline, and pinned open for macroscopic examination and for photographic documentation with a digital camera. The total gastric area (GA) and total ulcer area (UA) were analyzed by using Image Processing and Analysis in Java (ImageJ) from the National Institutes of Health (National Institutes of Health, Bethesda, MA, USA). Ulcer index (UI) scores were graded based on an arbitrary scale according to Zhou et al. [7]. The percentage of UA inhibition was calculated according to the following equation:

$$\%\;\mathrm{UA}\;\mathrm{inhibition}=\left(\frac{\mathrm{UA}\;(\mathrm{vehicle}\;\mathrm{group})\;-\;\mathrm{UA}\;(\mathrm{treated}\;\mathrm{group})}{\mathrm{UA}\;(\mathrm{vehicle}\;\mathrm{group})}\right)\;\times \;100\%$$

#### 2.5.5. Measurement of Oxidative Indicators

After photographing, the stomach segments were homogenized in ice-cold phosphate-buffered saline (PBS; pH 7.4), and 10% (*w*/*v*) homogenates were centrifuged at 3000× *g* rpm for 20 min. The supernatant was collected for determination of SOD, GSH, and CAT activity with enzyme-linked immunosorbent assay (ELISA) kits according to the manufacturer’s instructions. The absorbance was measured at 450 nm with a microplate reader.

#### 2.5.6. Measurement of ROS Activity, Lipid Peroxidation Products, and iNOS Levels

Stomach tissues were homogenized in PBS (pH 7.4) to obtain 10% (*w*/*v*) homogenates. Then, the suspensions were centrifuged at 3000× *g* rpm for 10 min and the supernatants were collected and used for measurements of ROS, MDA, and iNOS levels with ELISA kits. The absorbance values were measured at 450 nm using a microplate reader.

#### 2.5.7. Histopathological Analysis

Stomach tissues from each group were fixed in 10% formalin solution for 24 h, dehydrated, and embedded in paraffin. Tissue sections (5 μm) were cut and stained with hematoxylin and eosin (H&E) solution. Histopathological changes in the stomach, including edema, blood vessel changes, white blood cell infiltration, and glandular damage, were analyzed under a light microscope at 400× magnification.

#### 2.5.8. Statistical Analysis

Statistical analysis was performed using one-way analysis of variance (ANOVA) followed by Duncan’s post hoc test for multiple comparisons using the Statistical Product and Service Solutions (SPSS) software (version 20.0). All data are reported as the mean ± SEM. A *p* value of <0.05 was considered statistically significant.

## 3. Results

### 3.1. In Vitro Antioxidant Activity

The ORAC assay was used to measure the reduction in fluorescence caused by peroxyl radicals. As shown in Table 3, the ORAC of Phy-Blica-D was 12.95 ± 0.15 μM Trolox equivalents (TE)/µg of extract, as determined by a previously constructed Trolox standard curve. The results of the NBT dye reduction assay for Phy-Blica-D are shown in Table 3. Phy-Blica-D was shown to be an efficient superoxide anion scavenger with a 50% inhibition of superoxide anion radical (IC_50_) value of 85.44 ± 13.11 µg/mL. However, catechin as positive control had an IC_50_ value of 5.95 ± 0.46 µg/mL, which made it a better superoxide anion scavenger than Phy-Blica-D.

### 3.2. Effects of Phy-Blica-D on Ethanol-Induced Acute Gastric Lesions in Rats

**Figure 1:**
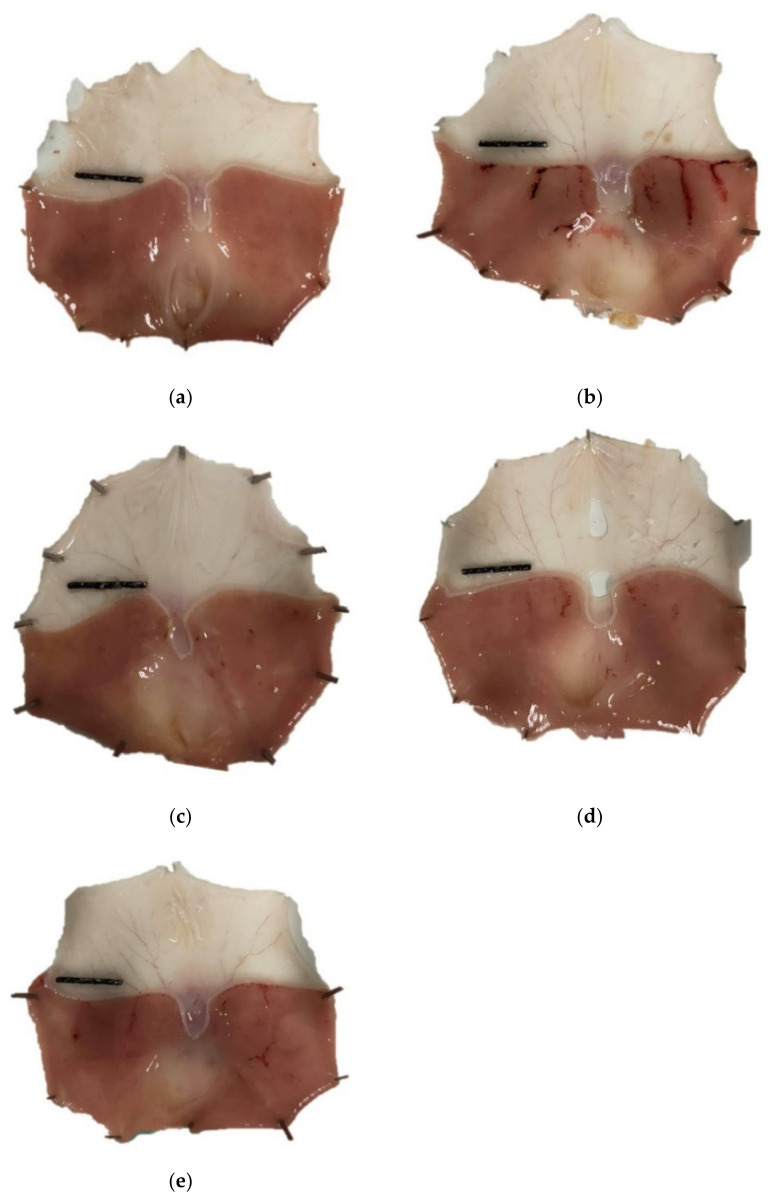
Effects of OMZ and Phy-Blica-D on the gross appearance of the gastric mucosa in ethanol-induced gastric ulcers in rats. (**a**) Normal group; (**b**) vehicle group; (**c**) OMZ group (20 mg/kg); (**d**) Phy-Blica-D (500 mg/kg) group; (**e**) Phy-Blica-D (1000 mg/kg) group. Scale bar = 1 cm.

As shown in Figure 1 and Table 4, after exposure to ethanol, severe hemorrhagic lesions that appeared as elongated bands parallel to the long axis of the glandular stomach with a UA of 0.60 ± 0.10 cm^2^ were observed in the vehicle group. In contrast, a marked decrease in the UA to 0.20 ± 0.08 cm^2^ (79.17% inhibition) was observed in the OMZ (20 mg/kg) positive control group. Treatment with different doses of Phy-Blica-D remarkably attenuated the severe injury caused by ethanol in the gastric mucosa. The best antiulcer effects were found in the 500 mg/kg Phy-Blica-D group, which gave the smallest UA (0.03 ± 0.01 cm^2^) and highest inhibition value (97.63%).

**Figure 2:**
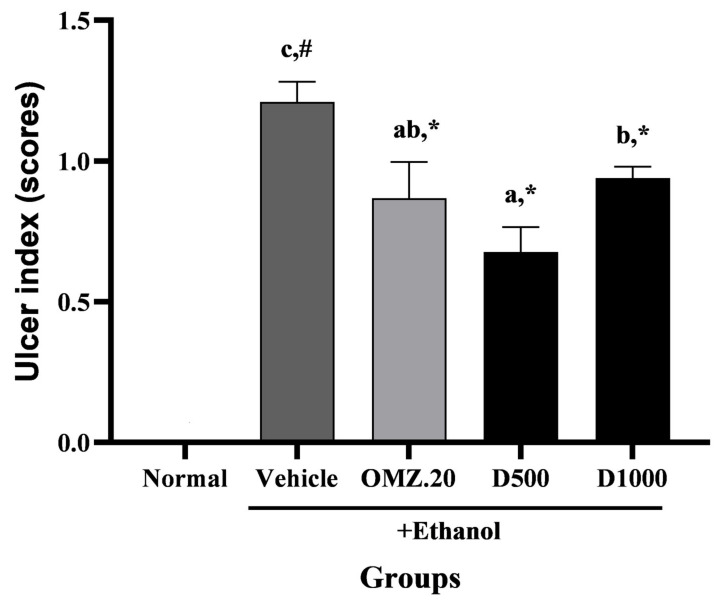
Effects of OMZ and Phy-Blica-D on the UI scores in rats exposed to ethanol. Data are expressed as the mean ± SEM (*n* = 6 per group) and were analyzed using one-way ANOVA and Duncan’s post hoc multiple-comparison test. * Significantly different from the vehicle control at *p* < 0.05. ^#^ Significantly different from the normal group at *p* < 0.05. Different letters (^a–c^) indicate significant differences (*p* < 0.05) between treatments determined by Duncan’s multiple-range test.

Oral administration of 80% ethanol induced the formation of gross lesions with a markedly moderate UI in the gastric lumens of rats. OMZ and both doses of Phy-Blica-D significantly reduced the gastric UI (Figure 2). However, the improvement in the gastric UI was more pronounced in the 1000 mg/kg Phy-Blica-D pretreatment group than in the 500 mg/kg Phy-Blica-D pretreatment group.

### 3.3. Effects of Phy-Blica-D on Gastric Juice Acidity

Phy-Blica-D treatment did not affect the pH value of gastric juice (Table 4). However, compared with the vehicle, OMZ significantly increased the pH.

### 3.4. Effect of Phy-Blica-D on the Levels of Oxidative Indicators

**Figure 3:**
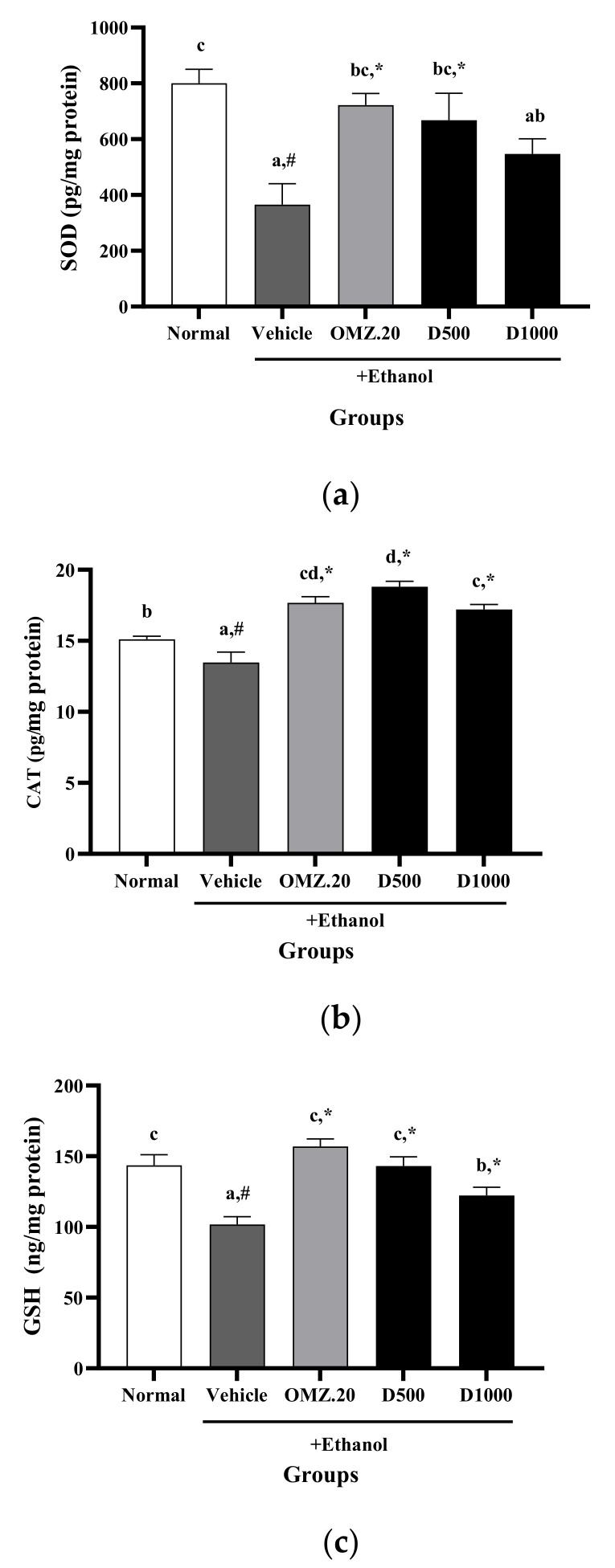
Effects of OMZ and Phy-Blica-D on the levels of SOD (**a**), CAT (**b**), and GSH (**c**) in the stomachs of rats exposed to ethanol. Data are expressed as the mean ± SEM (*n* = 6 per group) and were analyzed using one-way ANOVA and Duncan’s post hoc multiple-comparison test. * Significantly different from the vehicle control at *p* < 0.05. ^#^ Significantly different from the normal group at *p* < 0.05. Different letters (^a–d^) indicate significant differences (*p* < 0.05) between treatments determined by Duncan’s multiple-range test.

As shown in Figure 3, ethanol administration significantly (*p* < 0.05) decreased the GSH (101.82 ± 5.51 ng/mg protein), SOD (365.13 ± 75.14 pg/mg protein), and CAT (13.48 ± 0.72 pg/mg protein) levels compared with the normal control group (GSH: 143.53 ± 7.47 ng/mg protein; SOD: 800.44 ± 49.76 pg/mg protein; CAT: 15.11 ± 0.21 pg/mg protein). In contrast, compared with rats in the vehicle group, rats pretreated with OMZ and Phy-Blica-D exhibited remarkedly increased GSH, SOD, and CAT levels. Phy-Blica-D had the best effects in increasing GSH (143.03 ± 6.55 ng/mg protein), SOD (668.10 ± 96.73 pg/mg protein), and CAT (18.76 ± 0.39 pg/mg protein) levels when administered at a concentration of 500 mg/kg. These effects were similar to those in rats treated with OMZ. 

### 3.5. Effects of Phy-Blica-D on ROS Production

**Figure 4:**
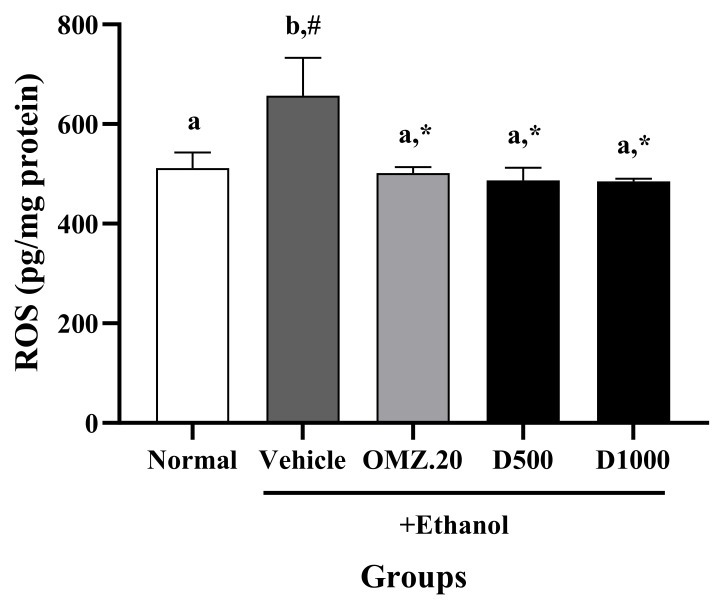
Effects of OMZ and Phy-Blica-D on the ROS levels in the stomachs of rats exposed to ethanol. Data are expressed as the mean ± SEM (*n* = 6 per group) and were analyzed using one-way ANOVA and Duncan’s post hoc multiple-comparison test. * Significantly different from the vehicle control at *p* < 0.05. ^#^ Significantly different from the normal group at *p* < 0.05. Different letters (^a,b^) indicate significant differences (*p* < 0.05) between treatments determined by Duncan’s multiple-range test.

As shown in Figure 4, a significant increase in ROS levels was found in the vehicle group (656.99 ± 75.76 pg/mg protein) compared with the normal group (511.60 ± 31.32 pg/mg protein), which was markedly reversed by OMZ and Phy-Blica-D treatment. While the group treated with 20 mg/kg OMZ (501.54 ± 12.39 pg/mg protein) showed reduced ROS levels, the rats treated with Phy-Blica-D at doses of 500 mg/kg (487.06 ± 25.14 pg/mg protein) and 1000 mg/kg (485.14 ± 4.89 pg/mg protein) showed the greatest reduction in ROS levels.

### 3.6. Effect of Phy-Blica-D on MDA Activity and iNOS Levels

**Figure 5:**
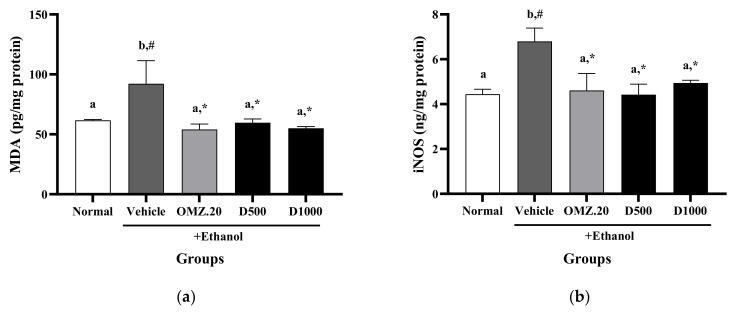
Effects of OMZ and Phy-Blica-D on the levels of MDA (**a**) and iNOS (**b**) in the stomachs of rats exposed to ethanol. Data are expressed as the mean ± SEM (*n* = 6 per group) and were analyzed using one-way ANOVA and Duncan’s post hoc multiple-comparison test. * Significantly different from the vehicle control at *p* < 0.05. ^#^ Significantly different from the normal group at *p* < 0.05. Different letters (^a,b^) indicate significant differences (*p* < 0.05) between treatments determined by Duncan’s multiple-range test.

Ethanol administration significantly increased the MDA activity (92.19 ± 19.29 pg/mg protein) and iNOS levels (6.80 ± 0.59 ng/mg protein) in gastric tissues compared to the normal control group (MDA: 61.71 ± 0.77 pg/mg protein; iNOS: 4.44 ± 0.22 ng/mg protein) (*p* < 0.05). In contrast, compared with vehicle treatment, treatment with 20 mg/kg OMZ led to obvious attenuation of the increase in the MDA activity (54.06 ± 4.53 pg/mg protein) and iNOS levels (4.61 ± 0.75 ng/mg protein) in the gastric tissue. Both doses of Phy-Blica-D (500 and 1000 mg/kg) also showed marked decreases in MDA activity and iNOS levels compared with the vehicle group (*p* < 0.05). There were no significant differences between the 500 and 1000 mg/kg Phy-Blica-D and OMZ groups (Figure 5a,b).

### 3.7. Histopathological Changes in Stomach Tissues

**Figure 6:**
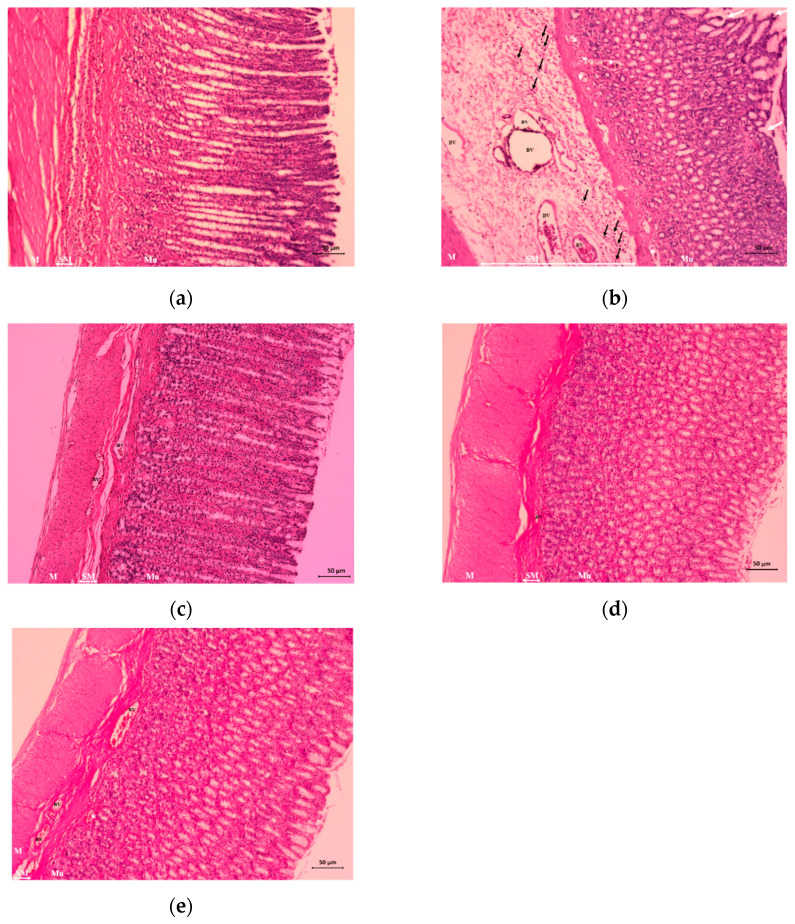
Effects of OMZ and Phy-Blica-D on histopathological lesions in gastric mucosa in rats exposed to ethanol (H&E staining, 400×) (*n* = 6 per group). (**a**) Normal group; (**b**) vehicle group; (**c**) OMZ group (20 mg/kg); (**d**) Phy-Blica-D (500 mg/kg) group; (**e**) Phy-Blica-D (1000 mg/kg) group. Abbreviations: Mu = mucosal layer, SM = submucosal layer, M = muscle layer, BV = blood vessel. The white arrows show broad erosion in the upper half of the mucosa, the black arrows indicate inflammatory cell infiltration, and the white asterisk indicates a high degree of hemorrhagic injury. Scale bar = 50 µm.

As shown in Figure 6, the normal group displayed normal columnar surface mucous and intact glandular cells (Figure 6a). Exposure of rats to ethanol produced extensive gastric mucosa damage and edema, dilated blood vessels, and inflammatory cell infiltration of the submucosal layer (Figure 6b). Pretreatment with OMZ completely protected the stomach mucosa, as indicated by the absence of edema and inflammatory cell infiltration (Figure 6c). Rats that received pretreatment with either dose of Phy-Blica-D exhibited comparatively better protection of the gastric mucosa, as indicated by reductions in the UA, submucosal edema, and inflammatory cell infiltration (Figure 6d,e).

## 4. Discussion

Phy-Blica-D is a traditional Thai herbal remedy that is used as a rejuvenating formula. It can reduce oxidative stress in vitro and has been regarded as safe in subacute toxicity studies in vivo [17]. Oxidative stress and excessive ROS levels are involved in several human diseases, such as cardiovascular, autoimmune, neurodegenerative, respiratory, and gastrointestinal diseases [39]. Gastric ulcers, another gastrointestinal disease, are caused by oxidative stress and are related to dysfunction in the antioxidant system [18]. Therefore, finding a new herbal remedy with highly effective antioxidant activity for the treatment of gastric ulcers has attracted considerable interest. Previous studies found that Phy-Blica-D has high radical-scavenging, antioxidant, and metal-chelating activities [17]. Data from the current work also demonstrated that Phy-Blica-D scavenged free radicals, including peroxyl radicals and superoxide anions. However, the gastroprotective effects of Phy-Blica-D remain uncertain. In this study, we first explored the gastroprotective effect of Phy-Blica-D against ethanol-induced gastric ulcers in rats for the possibility of its suppression of oxidative stress and stimulation of antioxidant enzymes in gastric tissue.

Ethanol-induced gastric ulcer models are commonly used to evaluate the pathogenesis of gastric ulceration and investigate the gastroprotective effects of various drugs and natural products [40]. Previous studies have suggested that ethanol ingestion leads to gastric ulcers, which are significantly associated with increased oxidative stress and increases in the levels of free radicals derived from oxygen, leading to a reduction in antioxidant enzyme activity [41]. Our findings were in line with those of previous studies indicating that 80% ethanol induces gastric ulcers by increasing ROS levels. The generation of high levels of ROS in gastric tissue plays a vital role in the formation of lipid peroxides, such as MDA, and is accompanied by antioxidative enzyme activity impairment in cells [42,43]. The results of the present study demonstrated that rats exposed to ethanol showed severe gastric lesions, increased free radical levels, and decreased levels of both antioxidative enzymes and non-enzymatic antioxidants. However, pretreatment with OMZ or Phy-Blica-D significantly decreased the free radical levels in rats with ethanol-induced ulcers. In addition, the levels of GSH, SOD, and CAT in the gastric tissues of rats treated with OMZ and Phy-Blica-D were significantly higher. Thus, the gastroprotective effects produced by Phy-Blica-D involve not only the reduction of oxidative stress, but also an increase in the antioxidant enzyme and non-enzymatic antioxidant levels.

ROS-activated lipid peroxidation is caused by a process in which free radicals interact with cell membranes to produce lipid peroxides, such as MDA. The level of MDA can indicate infiltration of neutrophils into gastric mucosal tissues [38,44]. In this study, the histopathological analysis showed abundant inflammatory cell infiltration into the damaged rat gastric tissues after ethanol exposure. Moreover, we found that MDA levels dramatically increased in gastric tissue exposed to ethanol without treatment. On the other hand, compared with the vehicle, pretreatment with OMZ and Phy-Blica-D significantly diminished MDA levels by approximately 40%. In addition, histopathological analysis demonstrated that the gastric tissue treated with OMZ and Phy-Blica-D had slight inflammatory cell infiltration. These results indicated that the properties by which Phy-Blica-D ameliorates gastric ulcers might be associated with its anti-lipid peroxidation properties by modulating inflammation.

A remarkable increase in NF-κB gene expression was found in ethanol-induced gastric ulcers. NF-κB is a transcription factor that binds to κB motifs in the promoters of target genes to induce the transcription of iNOS, COX-2, and inflammatory cytokines [45]. iNOS produces NO as a short-lived small molecule and plays a crucial role in the inflammatory processes. A previous study indicated that ethanol-induced gastric ulcers significantly increased NO levels and the expression of iNOS in gastric tissue, which stimulated inflammatory conditions [46]. The present study established that iNOS levels significantly increased in gastric tissue when only ethanol was administered. Conversely, the levels of iNOS were decreased by OMZ and Phy-Blica-D treatment. These results indicated that the improvement of gastric ulcers by Phy-Blica-D might be associated with its anti-inflammatory effects.

OMZ is widely used for the treatment of gastric ulcers because of its anti-inflammatory properties, antioxidant activity, and acid secretion suppressing ability; thus, it was used as a positive control in this study [47,48]. Our results showed that compared with the vehicle treatment, pretreatment with 20 mg/kg OMZ significantly increased the gastric pH value in rats. However, Phy-Blica-D treatment did not affect the gastric pH. Normally, acid secretion is suppressed by standard drugs, such as OMZ, through proton-pump inhibition [9]. Therefore, we concluded that gastric acid inhibition is unlikely to be a mechanism underlying the beneficial effects of Phy-Blica-D.

Previous studies have reported that Phy-Blica-D consists of 18 chemical compounds, including 6-galloylglucose, 1-O-galloylglycerol, fertaric acid, vanilpyruvic acid, (2S)-5,7,3′,4′-tetrahydroxyflavanone 6-C-glucoside, naringerin, agecorynin B, castavinol, chalconaringenin 2′-rhamnosyl-(1→4)-xyloside, beta-rhodomycin, sericoside, alliosterol 1-(4″-galactosylrhamnoside) 16-galactoside, licorice saponin A3, asparasaponin II, licorice saponin G2, betavulgaroside II, glycyrrhizic acid, and 6-gingerol [17]. Interestingly, some of the chemical compounds in Phy-Blica-D, such as naringerin [49,50,51], glycyrrhizic acid [52,53], saponins [54,55]. 6-gingerol [56,57], 1-O-galloylglycerol [58], and fertaric acid, have been shown to be strong antioxidants with gastroprotective effects [59,60]. Therefore, there is a possibility that the gastroprotective effects of Phy-Blica-D may result from the presence of these chemical compounds. However, other phytochemical compounds in Phy-Blica-D absolutely must be analyzed in the future to confirm their gastroprotective properties.

The 500 and 1000 mg/kg doses of Phy-Blica-D showed similar gastroprotective capacities. The UAs, pH values, percent inhibition of gastric ulcers, and ROS, MDA, and iNOS levels were not significantly different between these doses (*p* > 0.05). However, 500 mg/kg Phy-Blica-D showed higher levels of SOD, CAT, and GSH than the 1000 mg/kg dose. Moreover, the 1000 mg/kg doses of Phy-Blica-D showed a higher UI than that of the 500 mg/kg dose, but lower than that of the vehicle control. It is possible that the presence of some phytochemical compounds of the high dose had an irritating effect on the gastric mucosa. These results are scientific evidence for the collection of the appropriate dose, 500 mg/kg, in order to develop products for gastroprotection that reduce the side effects of highly concentrated Phy-Blica-D, which may be associated with irritation and increased gastric ulcers.

Therefore, the proper dose of Phy-Blica-D for gastroprotection is 500 mg/kg, as this dose of Phy-Blica-D exerted effects that were not significantly different from those of the standard drug, OMZ.

## 5. Conclusions

This study demonstrated that Phy-Blica-D has potential antioxidant activity in vitro and in vivo. Oral administration of Phy-Blica-D once daily for 7 days alleviated gastric ulcers induced by ethanol. The antiulcer effects of Phy-Blica-D not only markedly inhibited oxidative stress, but also increased the activities of GSH, SOD, and CAT, which reduced the lipid peroxidase MDA. Moreover, Phy-Blica-D pretreatment suppressed iNOS levels, which may reduce inflammation in gastric ulcers. Therefore, it is reasonable to believe that the gastroprotective effects of Phy-Blica-D may involve the suppression of oxidative stress and an increase in antioxidant activity.

## Figures and Tables

**Table 1:** Medicinal components of the traditional Thai polyherbal drug Phy-Blica-D (per 150 g formulation) and its phytochemical information.

No.	Scientific Names	Part Used	Proportion	Phytochemical Compounds
1	*Glycyrrhiza glabra* Linn.	Bark	47	Glycyrrhizin, liquiritic acid, glycyrretol, glabrolide, isoglaborlide, liquorice acid, liquiritin, liquiritigenin, hamnoliquiritin, neoliquiritin, etc. [18,19]
2	*Aegle marmelos* (L.) Correa ex Roxb.	Fruit	36	Aegeline, aegelenine, aegelinosides, marmelin, marmelosin, anhydromarmeline, marmelide, etc. [20]
3	*Phyllanthus emblica* L.	Fruit	18	Ascorbic acid, sesamine, phyllantidine, β-carotene, estradiol astragalin, ellagic acid, rutin, lupenone, kaempferol, pedunculagin, quercetin, phylianthin, etc. [21]
4	*Terminalia arjuna* Wight and Arn.	Fruit	11	Arjunic acid, arjunone, arachidic stearate, cerasidin, ellagic acid, fridelin, gallic acid, hentriacontane, methyl oleaolate, myristyl oleate, β-sitisterol, etc. [22]
5	*Terminalia bellirica* (Gaertn.) Roxb.	Fruit	11	Tainternilignan, thannilignan, flavones, anolignan B 5, gallic acid, β-setosterol, tannins, alkaloids, saponin, polysaccharides, steroid, belleric acid, galactose, chebulagic Acid, etc. [23,24]
6	*Cyperus rotundus* Linn.	Rhizomes	4	Sitosterol, glycosides, α-rotunol, betacyperone, β-selinene, camphene, cyperene, cyperenon, cyperol, cyperolon selinene, cyperotundone, linolenic acid, linoleic acid, oleic acid, rotundene, rotundenol, rotundone, polyphenols, etc. [25,26]
7	*Maerua siamensis* (Kurz) Pax.	Root	4	Cappariloside A, cappariloside B, glochidone, lupeol, chrysoeriol, cinnamic acid, vanillin, etc. [27]
8	*Terminalia citrina* Roxb. ex Fleming	Fruit	4	Triterpenes, flavonoids, tannins, lignan, terminalosides A–K, 2-epiterminaloside D, 6-epiterminaloside K, etc. [28]
9	*Piper retrofractum* Vahl.	Fruit	4	Caryophyllene, pentadecane, 1,4,7,-Cycloundecatriene, 1,5,9,9-tetramethyl-, Z,Z,Z, 8-heptadecene, Heptadecane, etc. [29]
10	*Zingiber officinale* Roscoe.	Rhizomes	4	Quercetin, zingerone, gingerenone-A, 6-dehydrogingerdione, β-bisabolene, α-curcumene, zingiberene, α-farnesene, etc. [30]
11	*Alpinia galanga* (L.) Willd.	Rhizomes	4	β-bisabolene, cis-α-bergamotene, β-sesquiphellandrene, 1,8-cineole, Chavicol, acetate, etc. [31]
12	*Solanum torvum* Swartz.	Fruit	4	Alkaloids, flavonoids, tannins, saponins, glycosides, oil, tocopherol/Vitamins E, B, C, etc. [32]
13	*Allium sativum* L.	Bulb	1	Allicin, E-ajoene, Z-Ajoene, 2-Vinyl-4H-1,3-dithiin, diallyl disulfide, diallyl trisulfide, etc. [33]
14	*Tinospora crispa* (L.) Miers ex Hook.f. and Thoms.	Stem	1	Borapetoside A-H, rumphioside A-C, columbin, tinocrisposide A-D, apeginin, diosmetin, genkwanin, d luteolin 4′-methyl ether 3′-glucoside, etc. [34,35]

**Table 2:** Experimental design. Phy-Blica-D was administered as a pretreatment for 7 days.

Group	Animals	Administered Substance(s)
Normal control	Untreated rats	Distilled water (1 mL/kg BW)
Vehicle control	Gastric ulcer rats	Distilled water and ethanol
Positive control	Gastric ulcer rats	OMZ (20 mg/kg) and ethanol
Experimental group	Gastric ulcer rats	Phy-Blica-D (500 mg/kg) and ethanol
Experimental group	Gastric ulcer rats	Phy-Blica-D (1000 mg/kg) and ethanol

**Table 3:** Peroxyl and superoxide anion radical scavenging properties of Phy-Blica-D.

Tested Extracts	Peroxyl Radical Trolox Equivalent (µM TE/µg of Extract)	Superoxide Anion Radical IC_50_ (µg/mL)
Phy-Blica-D	12.95 ± 0.15	85.44 ± 13.11 ^b^
Catechin	-	5.95 ± 0.46 ^a^

The results are presented as the mean ± SD; ANOVA and Duncan’s test. ^a,b^ Values in the same column with different superscripts are significantly different (*p* < 0.05).

**Table 4:** Effect of Phy-Blica-D on gastric lesions induced by 80% ethanol in rats.

Group	pH Value	GA (cm^2^)	UA (cm^2^)	% Inhibition
Normal	2.50 ± 0.29 ^a^	15.76 ± 0.46	0.00 ± 0.00 ^a^	-
Vehicle	2.25 ± 0.25 ^a^	16.08 ± 0.65	0.60 ± 0.10 ^#,c^	-
OMZ.20	5.25 ± 1.03 *^,b^	16.62 ± 0.74	0.20 ± 0.08 *^,b^	79.17 ± 7.98 ^b^
D500	2.50 ± 0.29 ^a^	16.80 ± 1.87	0.03 ± 0.01 *^,a,b^	97.63 ± 0.77 ^a^
D1000	2.75 ± 0.25 ^a^	16.28 ± 1.27	0.06 ± 0.04 *^,a,b^	97.42 ± 1.14 ^a^

Data are expressed as the mean ± SEM (*n* = 6 per group) and were analyzed using one-way ANOVA and a post hoc Duncan multiple-comparison test. * Significantly different from the vehicle control at *p* < 0.05. ^#^ Significantly different from the normal group at *p* < 0.05. GA, total gastric area; UA, total ulcer area; % inhibition, percent ulcer area inhibition. Different letters (^a–c^) indicate significant differences (*p* < 0.05) between treatments determined by Duncan’s multiple-range test.

## Data Availability

The data that support the findings of this study are available on request from the corresponding author.
